# William Michell Clarke, M.R.C.S., L.S.A., *Consulting Surgeon to the Bristol General Hospital*

**Published:** 1885-12

**Authors:** 


### Obituary.

### WILLIAM MICHELL CLARKE, M.R.C.S., L.S.A.,

*Consulting Surgeon to the Bristol General Hospital.*

We have to record with much regret the sudden and unexpected death of William Michell Clarke. The deceased gentleman died at his residence, 2 York Buildings, Clifton, on October 2nd. He had been somewhat indisposed for a few days, but his friends noticed nothing in his condition to excite alarm; indeed, he continued his professional work to the very last, and saw patients on the day preceding his death. Never of very robust strength, he was able by careful management to carry on one of the largest practices in Clifton and its neighbourhood, and his loss will be greatly felt by numerous patients and friends. He leaves a void in professional circles that will not be easily filled, and by none will his loss be more felt than by the members of the Bristol Medico-Chirurgical Society. Mr. Clarke was, in 1879, President of this Society, and read a most interesting address on "The Natural History of Disease." He was also a frequent contributor to the ordinary meetings, besides taking a deep interest in the general well-being of the Society.

Mr. Clarke may be looked upon as one of our most successful practitioners, and he owed his success entirely to his own individual efforts, and to thorough and conscientious work. His example may be taken as showing what may be accomplished in our profession without extraneous aid by a man who is thoroughly in earnest.

Mr. Clarke began his professional career by being apprenticed to a country Surgeon at Bodmin, his native town. He afterwards entered St. Bartholomew's Hospital, where he was known as a most industrious and painstaking student. After leaving St. Bartholomew's, he was appointed House Surgeon to the Bristol General Hospital, which at that time contained only about twenty beds, and was located in two or three ill-contrived houses. Here Mr. Clarke worked hard and well, and ultimately had the satisfaction of being Surgeon, and afterwards Consulting Surgeon, in the same Institution, when it had a local habitation worthy of its name. As a Surgeon, Mr. Clarke was most careful and discriminating, and had performed most of the great operations in Surgery; and although he was compelled by the rapid increase of his private practice to resign the active duties of Surgeon at the end of the comparatively short period of ten years, yet he continued his connection with the Hospital in the capacity of Consulting Surgeon, and was most regular in his attendance on the fixed operating days; his services as Consulting Surgeon being much appreciated by his acting colleagues.

In his private practice, which was rather more medical than surgical, he was successful more than ordinary men. Beginning practice first near the Hospital, he speedily moved up to Clifton, where the public and his medical friends soon recognized his merits; and especially was he favourably known to the late Drs. Symonds and Budd. A few years after settling in Clifton, Mr. Godfrey, who was one of his surgical colleagues at the Hospital, contracted typhoid fever and died; and Mr. Clarke succeeding to his practice, found himself, at a comparatively early age, one of the busiest men in Clifton.

Mr. Clarke had not only filled the office of President of the Bristol Medico-Chirurgical Society, but had also been President of the Bath and Bristol Branch of the British Medical Association. He was also formerly Lecturer on Medical Jurisprudence at the Bristol Medical School, and at the time of his death was one of the Governing Body of that Institution.

Few men were more unselfish with regard to acquiring patients; and in all his dealings with them, and also with the members of his own profession, he was honest, honourable, and straightforward, and was in consequence held in the highest esteem, as was testified (if such testimony were needed) by the large number of professional friends who attended his funeral.

